# Appreciation and implementation of the Krachtvoer healthy diet promotion programme for 12- to 14- year-old students of prevocational schools

**DOI:** 10.1186/1471-2458-11-909

**Published:** 2011-12-08

**Authors:** Kathelijne MHH Bessems, Patricia van Assema, Marloes K Martens, Theo GWM Paulussen, Lieke GM Raaijmakers, Nanne K de Vries

**Affiliations:** 1 NUTRIM School for Nutrition, Toxicology, and Metabolism, Department of Health Promotion, Maastricht University Medical Centre+, P.O. Box 616, 6200, MD Maastricht, The Netherlands; 2 ResCon, Rijswijkstraat 175, 1062, EV Amsterdam, the Netherlands; 3 TNO (Netherlands Organisation of Applied Scientific Research) Quality of Life, Postbus 2215, 2301, CE Leiden, the Netherlands; 4 CAPHRI School for Public Health and Primary Care, Maastricht University Medical Centre+, P.O. Box 616, 6200, MD Maastricht, the Netherlands

## Abstract

**Background:**

Krachtvoer is a school-based healthy diet programme, developed in 2001 and revised in 2007 to meet the needs of particular segments of the target population as well as a wider target group. The main aims of the present process evaluation of the revised programme were to examine student and teacher appreciation of the programme, completeness of and adherence to its implementation, and relations between appreciation and completeness of implementation.

**Methods:**

Data were collected among 22 teachers and 1117 students of 13 schools, using student evaluation forms, teacher logbooks, telephone interviews, and classroom observations.

**Results:**

Results indicate favourable levels of teacher and student appreciation for the programme in general and the revised elements. Girls, first-year students and students with more favourable dietary intakes particularly appreciated individual programme elements. Levels of completeness of implementation were high, but several teachers did not adhere to the intended implementation period. Some moderately strong relations were found between teacher appreciation and completeness of implementation scores.

**Conclusion:**

We conclude that the revisions have resulted in a programme that was appreciated well, also by the extended target group, and was implemented with a high degree of completeness. Teacher appreciation proved potentially important for completeness of implementation. We identified several aspects requiring improvement, indicating the importance of continued programme updates and repeated evaluation.

## Background

Unhealthy dietary habits, such as inadequate fruit consumption, skipping breakfast and a high consumption of saturated fats, are among the main life style factors associated with the majority of chronic diseases such as coronary heart disease, several types of cancer and type 2 diabetes, as well as with overweight and obesity [1,2]. Like other Western countries, the Netherlands is faced with increasing numbers of chronic diseases and cases of overweight and obesity, even among younger people [3,4]. In 1980, 6% of the boys and girls between the ages of 2 and 21 were overweight, and this percentage had increased to 14% in 2010 [5].

Dietary habits of youngsters become less healthy especially at the time of transfer from primary to secondary school, as they become more independent in their food choices. By the age of 13 years only 28% to 39% of the Dutch adolescents consume fruit on a daily basis [3]. A daily breakfast is consumed by 86% of the Dutch 12- to 13-year-olds, but grain products are consumed at breakfast by only 52% in this age group [6]. Snacks are eaten more than two times a day by 52% of the 12- to 15-year-olds [7]. This underlines the importance of health promotion activities to improve the dietary habits of these adolescents.

The Dutch Ministry of Health aims to stop the increase in chronic diseases and obesity, for instance by means of behavioural change programmes to achieve healthier lifestyles, including healthy dietary intakes [8]. Specific target groups of the Dutch policy are young people and people with lower socio-economic positions (SEP), since their lifestyles are less healthy than those of their peers with higher SEP [9-11].

Krachtvoer is a Dutch school-based healthy diet programme for first-year and second-year students of prevocational schools. Prevocational schools tend to have more students coming from families with lower SEP than students attending higher educational levels [12]. The programme consists of eight lessons, including fixed lessons and optional activities. The main aims are to increase the intake of fruit, to promote the daily consumption of a healthy breakfast, and to reduce the consumption of high-fat snacks.

The first version of the programme was launched and evaluated in 2001. The programme had some beneficial effects on students' dietary intakes, but also some undesirable effects among students with favourable pre-programme intakes [13]. It was appreciated quite well and was implemented with satisfactory levels of completeness and adherence. However, some improperly implemented or less appreciated programme elements clearly needed revision. Better guidance for teachers was recommended to improve programme implementation [14].

The programme was revised in 2007, to improve the weaker elements in the evaluation, to modernize the design of the programme materials, and to meet the specific needs of particular segments in the target population. This paper describes the process evaluation of the revised programme.

Process evaluations are recommended to evaluate a programme's compatibility with the target population and the fidelity of programme implementation [15,16]. Two aspects of fidelity of programme implementation include adherence (is the programme implemented as intended by its developers?) and completeness (what proportion of activities have actually been implemented?). The first goal of the current study was to examine the appreciation of the programme among students and teachers. More specifically, we investigated if the programme revisions had resulted in greater programme appreciation, also by subgroups based on gender, year (first or second) of the class, educational level, SEP, ethnicity and dietary consumption before the start of the programme. The second goal of the study was to assess the completeness of implementation of specific programme elements and the programme adherence. Finally, we examined the relation between programme appreciation and programme completeness. We wanted to find out if the qualitative findings of the first process evaluation, which indicated that programme appreciation among teachers might be related to the completeness of implementation [14], could be confirmed.

## Methods

### The revised Krachtvoer programme

The first version of Krachtvoer had been developed using key components of the Intervention Mapping procedure [17], including the use of available empirical and theoretical knowledge, and additional data collection among users. For the revised version a revision plan was developed based on the evaluations of the first version of the programme [13,14], a new literature study and interviews with researchers of other health promotion programmes. The revised programme was carefully pretested among teachers and students.

The programme consists of eight lessons intended to be taught by a school teacher biology (subject which is focused on technical knowledge, e.g. on nutrients) or care (subject which is focused on practical knowledge and skills, e.g. cooking skills). Lessons are intended to be taught in a specific order within six to ten weeks. The programme includes fixed lesson parts (i.e. lesson parts with a fixed protocol) and optional activities. Programme activities can be taught in single (45 to 50 minutes) or two-period lessons (90 to 100 minutes), and may be combined with regular teaching materials.

The programme builds on the three phases of self-management theory [18], successively aimed at raising awareness about personal dietary intakes, proposing solutions for not meeting the Dutch dietary guidelines, and setting personal goals for dietary improvement [19]. Additionally, it uses insights from the Theory of Planned Behaviour [20], the Attitude-Social influence-Self-Efficacy Model [21], and action planning literature [22]. Some examples of theory-based methods we used are goal setting and feedback.

The main instrument is the student work book, which includes information sheets (e.g. with information about the national recommendations for fruit intake), question sheets (e.g. with questions on students' own fruit intake), and activity sheets (e.g. describing a fruit shake preparation activity). The programme is supported by a teacher manual, posters, postcards, a lunchbox with a flyer and healthy food items, a magazine, a recipe contest, a website (with information, knowledge tests, and recipes), and a take-home bag with a note pad, healthy products and a newsletter with tips and recipes. Many of the materials feature three cartoon characters related to the targeted behaviours. A programme overview is given in Table 1.

**Table 1:** Overview of the Krachtvoer programme

Phase	Lesson number and topic	Content in short	Supportive materials
1	Prior to the lessons	• Students receive one of three different postcards designed to introduce the programme name, the three cartoon characters representing the three topics, and the programme website.	• Postcards • Posters
		• Posters matching the design of the postcards are put up in the classroom.	
	0. Nutrients	Pre-programme optional lesson	• Workbook
		• A general lesson on nutrients (fats, saturated fats, carbohydrates, proteins, fibres, minerals and water), their functions in the human body, and food products containing these nutrients.	
	1. Nutrition, foods and health	Fixed lesson	• Workbook
		• Different types of knowledge (e.g. theoretical, applied) are tested in a quiz about fruit, breakfast and low-fat snacks, to make students enthusiastic about the programme. The quiz can be done at home with the parents as well.	• Lunchbox
		• Students receive a lunchbox with a flyer and three healthy food items representing the three topics of fruit, breakfast and healthy snacking.	
	2. Fruit and fruit juices	Fixed lesson	• Workbook
		• Photos of fruit juices and product labels are used to help students distinguish fruit juices from sugared fruit juices.	• Website
		• Knowledge about the differences between fruit juices and other juices is tested with a fruit juice test in the workbook or on the website.	
		• Writing down their own fruit consumption and being provided with information about the national recommendations makes students aware of their own fruit intakes compared to the recommendations.	
	3. Breakfast and snacks	Fixed lesson	• Workbook
		• Writing down their own breakfast consumption makes students aware of their own breakfast intakes. Students can compare their own breakfast with the national recommendations for a healthy breakfast consisting of five food groups(the 'disc of five').	
		• Writing down their own snack consumption makes students aware of their own snack intakes. An overview is given of low- and high-fat snacks, which they can compare to the snacks they regularly consume.	
2	4 (First part) Barriers to healthy eating	Fixed lesson	• Workbook
		• Students answer questions on personal reasons for meeting or not meeting the dietary recommendations on fruit, breakfast and snack intakes. Students give each other tips on healthy eating.	• Take-home bag
		• Students receive a take-home bag with a notepad, healthy products, a newsletter with tips and recipes and a notepad to involve their parents.	
	4 (second part) to 7 (first part) Skills and knowledge	Optional activities (teacher can choose one or two)	• Workbook
		1. National recipe contest: students participate in a national recipe contest.	• Website
		2. Magazine: students work with a magazine offering information, tests, puzzles, a horoscope, role model stories and healthy recipes.	• Magazine
		3. Website: students visit the Krachtvoer website to read information, to do a snack test (i.e. distinguish high- and low-fat snacks) and knowledge test (e.g. practical and theoretical knowledge about fruit, breakfast and snacks), and send e-cards.	
	5 Food exposure	Optional activities (teacher can choose one)	• Workbook
		1. Taste testing: students judge products by tasting, smelling, and looking at (unfamiliar) fruit, breakfast products and healthy snacks.	• Fruit juice, breakfast and low-fat snack products
		2. Fruit tasting: students bring fruits from home to school and taste them together.	
		3. Preparing a fruit shake: students prepare a healthy tasty fruit shake.	• Products required to prepare a fruit shake
	6 Advertisements	Fixed lesson	• Workbook
		• Advertising tricks are discussed and tricks are applied in an advertising poster produced by students.	
3	7 (second part) Personal action plans	Fixed lesson	• Workbook
		• Students use a programme on the website to generate a personal action plan (what, when, where) to improve their fruit, breakfast or snack intake during the next week.	• Action plan computer program
	8 Evaluation of personal plan	Fixed lesson	• Workbook
		• The implementation of the action plans, difficulties encountered and possible solutions are discussed in class.	

Compared to the first version, the revised programme aims to more fully meet the needs of specific student subgroups, through the addition of tips to the teacher manual on the use of different strategies for students of the lower educational subtrack (i.e., strategies that are more active or interactive and more practical, and less based on individual reading and information processing: e.g. a quiz which is normally filled in individually by students can also be used as an active contest in which the teacher reads the questions out loud and students go sit in a "true" or "false section" of the classroom); through the addition of information on foods and dietry habits from other cultures for students of non-Dutch ethnicity (e.g. discussing the Ramadan); and through the inclusion of assignments for students who are already eating a healthy diet (e.g. by including assignments requiring them to give tips to others about healthy eating). In addition, the lay-out, size and composition of the materials were updated to meet the expectations of the current group of students (e.g. using full-colour materials). Specific changes to the lessons based on the findings of the first evaluation study included the addition of a pre-programme optional lesson with information on nutrients (lesson 0). In addition, three lessons on awareness of one's own dietary intakes from the previous version were combined into two lessons (lesson 2 and 3), the content of the lesson on advertisements was changed (watching television commercials and reflecting on its effects was replaced by preparing an advertisement poster and including advertisement tips) (lesson 6) and the lesson on formulating goals was substituted by an action plan computer program (lesson 7). Lastly, a dissemination strategy was developed, consisting of a handbook and a training course for health promotion professionals, to help them recruit teachers to work with the programme, organize a two hour kick-off meeting for the teachers, advise the teachers during programme implementation, evaluate the implementation and contribute to programme continuation in the schools [19].

### Data collection and measures

The current study was part of an RCT. The study was exempt from ethical review according to the Dutch review system [23]. Multiple data collection methods were used for the process evaluation, viz., student evaluation forms, teacher logbooks, classroom observations, and telephone interviews with teachers. Thirteen schools with 53 classes and 1117 students participated in the study. Students were invited to complete the student evaluation forms in the classroom directly after programme implementation. If students objected they were allowed to do another task during the lesson. All 22 teachers who taught the Krachtvoer programme were asked to complete the teacher logbook directly after each lesson. Five randomly selected teachers were approached and asked to give permission for the researcher to observe one to four of their lessons, and six other teachers were contacted for a telephone interview about adherence to the programme directly after implementation.

Student appreciation was measured by the student evaluation forms. One item assessed overall programme appreciation by asking the students to rate the programme on a scale of 1 to 10. In addition, students were asked to allocate separate ratings to five supportive programme materials (the lunchbox, take-home bag, postcards, posters, and website), and three fixed lessons and five optional activities which had undergone major revisions. Students only answered questions on programme elements which had actually been implemented by their teacher.

Teacher appreciation was measured by means of the teacher logbook, completed after each lesson. Mean total appreciation scores were calculated for the fixed lessons (or components thereof), the optional activities, and the supportive materials.

Completeness of implementation was also assessed by means of the teacher logbooks. The numbers of fixed lessons (or components thereof), optional activities, and supportive materials actually implemented or used were assessed with questions asking whether each of these elements had been implemented. If teachers had not taught a specific lesson or optional activity, they were invited to clarify their reasons for not doing so. Completeness of implementation of five fixed lessons (or components thereof) and four optional activities was calculated as the sum of activities that teachers had ticked as implemented on a list of all proposed activities in each fixed lesson or optional activity, divided by the total number of possible activities in each lesson or optional activity (range 0 to 1). These scores could not be calculated for two of the lessons and three optional activities, since they consisted of one activity only. Total mean completeness scores for fixed lessons and optional activities were calculated as well.

Adherence to the programme was measured by two items in the teacher logbook, i.e., whether the proposed order of the lessons had been adhered to (yes/no) and how long the implementation period had lasted. Teachers were invited to clarify any deviations from the proposed programme. Additionally, a researcher completed structured forms on implemented activities, additional materials used and teaching procedures during the classroom observations. Finally, deviations from the intended programme implementation were discussed in the telephone interviews with teachers.

### Student and teacher characteristics

Additional data were available on background characteristics of the students, viz. age, gender, country of birth of both parents and postal code of the home address. Ethnicity was defined as non-native (non-Dutch) if at least one of the parents had been born abroad [24]. Postal code was used as an indicator of the neighbourhood's SEP [25]. School-related characteristics included whether students were in the first or second year, the educational level of the class (lower [second subtrack] or higher [third and fourth subtracks]). Additionally, pre-programme food frequency data were derived from items in validated questionnaires [26,27], viz. the number of pieces of fruit eaten per day, the number of times a day that high-fat snacks were consumed and the number of days a week on which breakfast was consumed. Available teacher characteristics included gender, teaching experience, and subjects taught.

### Statistical analyses

Statistical analyses were conducted with PASW Statistics 17 (SPSS Inc. Chicago, IL).

Descriptive statistics were used to analyse participant characteristics and scores on outcome variables. Subgroups of students based on general programme appreciation and appreciation of specific programme elements were distinguished using backward regression analyses correcting for gender, year (first or second), educational track, SEP, ethnicity, and food intakes, using a 0.05 significance cut-off point. Pearson's correlations were used to explore possible relations between mean appreciation and implementation scores. In view of the small number of teachers in the study, we used a significance cut-off point of 0.10.

Qualitative data from the classroom observations were coded (in terms of programme adherence). Data from the telephone interviews were analysed by recording the interviews, preparing transcripts and coding these (in terms of programme adherence) with the NVIVO 8.0 qualitative data documentation and analysis software (QSR International, Doncaster, Australia). All codes were checked by a second researcher. Final summaries were written based on consensus about the codes between the two researchers.

## Results

### Response and participant characteristics

In the 13 schools that participated, 89 of the 1117 students did not complete the student evaluation form. The reason for this non-response as reported by the teachers was that these student had been absent from the lesson in which the evaluation form was completed. The final sample consisted of 1028 students. Around half of the students were male (51.2%). Most students were of Dutch origin (79.2%), attended the second year (64.7%), and were in the higher educational subtracks (77.3%). The students' SEP was comparable to the national mean (Table 2).

**Table 2:** Participant characteristics (*n *= 1028)

	Mean (SD) or %
Gender (%)	
Boys	51.2
Girls	48.8
Year (%)	
First	35.3
Second	64.7
Educational track (%)	
Lower subtrack of prevocational education	22.7
Higher subtracks of prevocational education	77.3
Ethnic status (%)	
Both parents born in het Netherlands	79.2
One or both parents born abroad	20.8
Mean age in years (SD)	13.0 (0.8)
Socio-economic position score based on postal code area (scale 4 to -4, low to high)	-0.04(0.86)

The majority of the 22 teachers who implemented the lessons were female (*n *= 13). The subjects they taught were biology (*n *= 5), care (*n *= 8) or both (*n *= 9). Their average teaching experience was 11 years (SD = 9 years). Seven schools had only one teacher teaching the Krachtvoer lessons, while the six other schools used two to five. The teacher logbook was fully completed by 18 teachers (81.8%). All non-respondents (two males, two females) worked at the same school, from which only one teacher logbook was received. The reason for this non-response was considered to be communication problems at the school. The five randomly selected teachers who were approached for classroom observations were all willing to participate, as were the six teachers who were approached for telephone interviews.

### Student and teacher appreciation

On average, the students rated the programme as 7.3 (SD = 1.3) out of 10. Table 3 shows that the teachers' total mean appreciation scores for the fixed lessons, the optional activities and the supportive materials were 7.0, 7.5 and 7.4, respectively. The most appreciated lessons and optional activities by students and teachers were food tasting and preparation activities. Teachers as well as students gave the lowest score to the evaluation of the personal plans. The supportive materials most appreciated by teachers and students were the lunchbox and take-home bag. The largest difference between student and teacher appreciation was found for the website, which was rated as 6.7 by the students and 7.7 by the teachers. Appreciation of the lessons that had undergone major changes (lessons 6, 7, and 8) and the added pre-programme optional lesson was comparable to that of the other lessons (average scores between 6.9 and 7.6 for teachers and between 6.5 and 7.2 for students). The subgroup analyses revealed that the overall appreciation of the programme was higher among girls, first-year students, students in the lower educational subtrack, students with lower SEP, students of non-Dutch origin and students with more favourable fruit and snack intakes (Table 4). As regards the specific programme elements, higher appreciation rates for the majority of elements were found among girls, first-year students and students with more favourable dietary intakes.

**Table 3:** Student and teacher programme appreciation and completeness of implementation of the programme elements

Fixed lessons, optional activities and supportive materials Lesson		Mean score student programme appreciation (SD)^1^; valid n		Mean score teacher programme appreciation (SD); valid n		Mean score teachers' completeness of implementation of programme elements (SD)^2^; valid n(%)	
	**Fixed lessons**						
	**(or parts thereof)**						
1	Nutrition, foods and health	-		7.0 (1.3);	n = 18	-	n = 18 (100%)
2	Fruit and fruit juices	-		7.0 (0.9);	n = 18	0.78 (0.21);	n = 18 (100%)
3	Breakfast and snacks	-		7.2 (1.0);	n = 18	0.64 (0.25);	n = 18 (100%)
4	Part 1: Barriers to healthy eating	-		7.0 (1.0);	n = 15	0.75 (0.27);	n = 15 (83%)
6	Advertisements	7.2 (1.8);	n = 385	7.5 (1.3);	n = 13	0.56 (0.25);	n = 15 (83%)
7	Part 2: Personal action plans	6.9 (1.9);	n = 348	7.6 (1.5);	n = 12	0.47 (0.30);	n = 15 (83%)
8	Evaluation of personal plans	6.5 (1.7);	n = 361	6.9 (1.5);	n = 10	-	n = 11 (61%)

**Total**				**7.0 (0.7)**		**0.70 (0.17)**	

	**Optional activities**						
0	Nutrients	-		7.0 (0.7);	n = 15	0.77 (0.20);	n = 15 (83%)
5	Taste testing	8.2 (1.5);	n = 765	8.1 (0.8);	n = 17	0.88 (0.22);	n = 17 (94%)
5	Fruit tasting	-		8.1 (1.1);	n = 4	-	n = 4 (22%)
5	Preparing a fruit shake	8.0 (1.6);	n = 421	8.2 (1.2);	n = 5	0.90 (0.22);	n = 5 (28%)
4-7	National recipe contest	7.2 (1.6);	n = 699	7.2 (1.5);	n = 16	-	n = 12 (67%)
4-7	Magazine	6.7 (1.6);	n = 700	7.4 (1.2);	n = 17	0.61 (0.26);	n = 14 (78%)
4-7	Tests on website	6.9 (1.5);	n = 679	7.4 (1.3);	n = 16	-	n = 13 (72%)

**Total**				**7.5 (0.7)**		**0.83 (0.09)**	

	**Supportive materials**						
	Lunchbox	7.6 (1.4);	n = 799	8.0 (1.7);	n = 18	-	n = 18 (100%)
	Take-home bag	7.4 (1.6);	n = 671	7.6 (1.6);	n = 18	-	n = 18 (100%)
	Postcards	6.7 (1.5);	n = 612	7.0 (1.3);	n = 18	-	n = 18 (100%)
	Posters	6.8 (1.5);	n = 587	6.7 (1.2);	n = 18	-	n = 18 (100%)
	Website	6.7 (1.7);	n = 618	7.7 (1.2);	n = 16	-	n = 16 (89%)

**Total**				**7.4 (1.1)**			

^a^A scale from 1 - 10 was used for appreciation scores. A higher score indicated higher appreciation. No appreciation data were available on the items indicated as -

^b^Some of the lessons and optional activities consisted of one activity, so no lesson or optional activity completeness scores were available for elements indicated as -

**Table 4:** Students' characteristics related to overall programme appreciation and programme element appreciation

	Gender(girls = 0, boys = 1)	Year (first- = 0, second = 1)	Educational track (low = 0, high = 1)	SEP(4 to -4, low to high)	Ethnicity(Non-Dutch = 0, Dutch = 1)	Fruit intake	Breakfast frequency	Snack frequency
**Overall programme appreciation Lessons**	-0.08*	-0.09*	-0.24***	0.09*	-0.090**	0.07*	-	-0.07*
Advertisements	-0.17***	-0.12**	-	-	-	-	0.12**	-
Action plan computer program	-0.19***	-0.16***	-	-	-	0.10*		-0.09*
**Optional activities**								
Taste testing	-	-	0.09**	-	-	-	-	-
Preparing a fruit shake	-	-	-	-	-	-	-	-
Recipe contest	-0.16***	-0.23***	-	-	-	-	-	-0.78*
Tests on website	-	-0.11**	-	-	-	-	-	-0.08*
Magazine	-0.11**	-0.12**	-	-	-	-	-	-0.10**
**Supportive materials**								
Lunchbox	-	-0.18***	-	-	-	-	0.09*	-
Take-home bag	-	-0.09*	-	0.09*	-	-	-	-
Postcards	-0.85*	-0.11**	-	-	-	-	-	-
Posters	-0.14**	-0.18***	-	-	-	-	-	-
Website	-0.13***	-0.13***	-	-	-	-	-	-

***P < 0.001, **P < 0.01, * *P *< 0.05

### Completeness of programme implementation

Lessons 1, 2, and 3 were implemented by all teachers, while the final lesson (evaluation of personal plan) was implemented by the smallest number of teachers (*n *= 11) (Table 3). The most frequently implemented optional activities were the taste testing, the pre-programme activity on nutrients and the magazine activity. Few teachers implemented the fruit shake preparation activity (*n *= 5) or the fruit tasting activity (*n *= 4), which were alternatives to the taste testing activity. Of the five supportive materials, the website was the only one not used by all teachers (two did not use it). On average, teachers implemented 6.1 (SD = 1.1) of the 7 fixed lessons (or components thereof) (range 4-7) and 4.6 (SD = 1.38) of the 7 optional activities (range 2-7).

The total mean completeness of implementation scores for the fixed lessons and the optional activities were 0.70 and 0.83, respectively. The lowest mean score was found for the action plan computer program (mean = 0.47). The fruit shake preparation activity was most completely implemented (mean = 0.90).

Reasons for skipping certain learning activities were mostly time-related, due to competing other events/activities (such as project weeks, national holidays) (*n *= 11), especially for the final lessons. Some teachers had postponed lessons until a later moment in the same school year (*n *= 4). Practical issues were also mentioned, such as lack of cooking or computer facilities (*n *= 4) and food items being past their use-by date due to delayed programme implementation (*n *= 1). All other reasons were mentioned by only one or two teachers; they included loss of students' enthusiasm about the programme, and activities being considered irrelevant (e.g. students were already aware of their own dietary intakes).

### Programme adherence

The teacher logbooks revealed that the intended order of the eight lessons was followed by all but one teacher, who postponed one lesson to the end of the programme. On average, teachers spent 8.4 weeks teaching the programme (range 1.5 to 16.0 weeks). Ten of the 18 teachers had implemented the programme over the intended implementation period. Time allocated for the lessons was considered sufficient by the majority of teachers. Some deviations from the intended programme were found, such as letting students work on the programme individually (as homework) instead of in groups, using too many additional materials, technical problems with the action plan computer program, and altering the fruit shake recipe by adding sugar. Some teachers were faced with problems keeping order in class.

### Relations between completeness of programme implementation and student and teacher appreciation

Teacher appreciation of the lessons was significantly positively correlated with teacher appreciation of the optional activities and the number of optional activities implemented (Table 5). Teacher appreciation of the supportive materials was significantly positively correlated with the number of lessons and optional activities implemented. A significant negative relation was found between the number of optional activities implemented and the completeness of lesson implementation.

**Table 5:** Correlations between total scores for programme appreciation and completeness of implementation (*n *= 18)

		Appreciation			Implementation			
		Teachers' lesson appreciation	Teachers' optional activity appreciation	Teachers' supportive material appreciation	Number of lessons implemented	Number of optional activities implemented	Lesson completeness	Optional activity completeness
**Appreciation**	Students' overall programme appreciation	-0.26	-0.19	0.09	0.33	0.30	-0.04	-0.23
	Teachers' lesson appreciation	1	0.80***	0.37	0.16	0.45*	0.36	-0.01
	Teachers' optional activity appreciation		1	0.13	0.29	0.26	0.10	-0.09
	Teachers' supportive material appreciation			1	0.55**	0.51**	0.04	-0.02
**Completeness of implementation**	Number of lessons implemented				1	0.23	0.01	-0.18
	Number of optional activities implemented					1	-0.40*	-0.18
	Completeness of lessons						1	-0.11

***P < 0.001, **P < 0.05, *P < 0.10

## Discussion

The aim of the present study was to investigate the appreciation of the Krachtvoer healthy diet promotion programme among teachers and students, as well as the completeness of implementation and adherence to the programme, and the relation between programme appreciation and the level of completeness of implementation.

Programme appreciation among students and teachers was satisfactory. Compared to the findings of the process evaluation of the first version of the programme [14], appreciation of those programme elements that had undergone major revisions was now comparable to that of the unchanged elements, indicating an increase in the intrinsic programme quality. Programme elements which included the preparation, tasting, or distribution of foods were appreciated most, as has also been reported by others [28,29]. The inclusion of such activities is recommended because of high appreciation rates and the potential contribution to behavioural change.

Our results on appreciation scores in subgroups of students show that the programme revisions were successful in making the programme appeal to a wider target group, including students of non-Dutch ethnicity, students attending a lower educational subtrack and students with more favourable dietary intakes. Girls were more positive about more than half of programme elements than boys, though this had not been intended by the developers. Although a recent comparable study [29] found no gender differences in the appreciation of a healthy diet programme among 10- to 12-year olds, previous studies found greater involvement in nutrition issues and more nutrition knowledge among girls and young women [30,31], which may explain the higher appreciation of our programme among girls. Our finding that first-year students appreciated almost all programme elements better than second-year students indicates how important it is that programmes for youngsters are made age-specific, although appreciation among second-year students was still acceptable. Students with more favourable dietary intakes appreciated half of the programme elements better than the other students. It is plausible that those who regard health as important are more inclined to appreciate health promotion initiatives and also show more healthy nutrition behaviour. The subgroup differences we found provide clues as to how a programme can be made appealing to specific subgroups. However, developing different programme elements and materials for specific subgroups may lead to higher costs and should therefore only be considered if the appreciation among some subgroups is unsatisfactory. Since programme appreciation was satisfactory in all subgroups in our study, our results do not imply a need for changes to the Krachtvoer programme at this point in time.

The number of programme elements implemented can be regarded as satisfactory, with 6 of the 7 fixed lessons (87%) and almost 5 of the 7 optional activities (65%) implemented. Although different measures were used in previous studies, which complicates comparisons, the implementation rates found in the present study match those of other school-based programmes, which ranged from 48% to 93% [29,32-36]. The only study which reported comparable measures of completeness of lesson implementation (in terms of activities per lesson) showed a slightly higher rate of 90% (similar to a score of 0.90 for our measure) [37], compared to our average rate of 0.70 for fixed lessons and 0.83 for optional activities.

In line with the findings of the evaluation of the first programme version, the present results show that the final two lessons had the lowest scores for appreciation and completeness of implementation. This may have been caused by aspects relating to the lessons as such, such as technical problems with the action plan computer program and the need to book a computer room for lesson 7, and the reflective nature of lesson 8. Logistical issues and class scheduling have been reported as barriers in other studies as well [37,38], and reflective lessons require more complex teacher skills. The low scores could also be due to the fact that the lessons were implemented at the end of the programme. Lower implementation rates of the final lessons have also been reported by others [14,33,34,36,38]. This may be inherent to implementation, possibly because students and/or teachers grow tired of a topic after spending much time on it, or because of time pressure, since other topics need to be taught as well. Time constraint was also reported as a barrier in other studies [34,37-39]. Lower implementation rates towards the end of a programme should therefore be taken into account by programme developers, by including essential programme elements earlier on in a programme.

With regard to adherence, no problems were reported with the order of the lessons, which is an improvement compared to the evaluation of the first version [14]. Almost half of the teachers did not implement the lessons within the intended time period. No corresponding data are available from the first trial. The finding could be explained by the fact that the lessons were taught in a busy period of the year, which may be less of a problem if teachers can decide for themselves when to implement the lessons. The classroom observations and telephone interviews revealed some other specific deviations from the teacher manual. The data collected in this study do not allow us to assess how frequent these deviations were, what caused them and how they impacted on the programme's results. However, it is obvious that the dissemination strategy should include specific strategies to deal with these adherence issues.

The final goal of our study was to explore the relation between programme appreciation and completeness of implementation. Three moderately positive correlations between teacher appreciation and the number of lessons and optional activities implemented indicate that programme appreciation by the teachers might be an important determinant of implementation. This supports the idea that health promotion programmes should be developed and continually revised in close collaboration with teachers in order to meet the needs of students and teachers and to ensure that the programme fits changing socio-political contexts (e.g. national dietary guidelines or changes in the educational system). The moderately negative relation between the number of optional activities implemented and the completeness of lesson implementation (in terms of activities per lesson) indicates that some teachers may have favoured quantity over quality. This issue needs to be dealt with in the dissemination strategy.

Some strengths and limitations of our study remain to be addressed. With regard to programme appreciation we found that programme appreciation by students and teachers has not often been examined this thoroughly in process evaluation studies, but proved to offer added value. It indicates that students from the expanded target group also appreciated the programme well and provides information about programme elements that are most eligible for improvement, either in general or for specific subgroups. Our findings can help others develop methods and strategies suitable for specific subgroups. We used more extensive appreciation measures for teachers than for students, since extensive measures were considered infeasible for students. This complicates comparisons of appreciation rates between students and teachers. With regard to completeness of implementation only a few studies have measured completeness of implementation of specific programme elements, which is a strength of our study, making it more specific. A limitation is that some teachers implemented the lessons in multiple (up to eight) classes, but teacher logbooks were only filled in once by each teacher. Variations between classes taught by one teacher were therefore not accounted for. Also, adherence to implementation was not measured very thoroughly. Still, the logbooks probably did not influence students' reactions the way observations by researchers might do. Lastly, we assume that teachers who participated in the study were thereby induced to teach the programme with higher levels of completeness and adherence as they had to record their activities in a logbook. Previous research has found that research participation is associated with higher levels of fidelity [40,41].

Our study has some implications for further research. Although we think that this study represents a step forward regarding the assessment of programme appreciation and completeness of implementation, further improvement of and agreement on measures is necessary. A complicating factor is that process evaluation measures need to be tailored to specific interventions. A promising but time-consuming method for measuring implementation quality is the use of video-recorded observations, which was applied in a study by Johnson and colleagues [42]. Further quantitative research is needed to investigate relations between appreciation by target group members and programme implementers, completeness of programme implementation and programme adherence.

## Conclusions

Our findings show that the revisions introduced in the Krachtvoer programme have resulted in a programme that was well appreciated, also by the expanded target group. It was implemented to a high degree of completeness. Nevertheless, this second process evaluation study again revealed several points that could be further improved, showing the importance of continued programme updates and repeated evaluation.

## Competing interests

The authors declare that they have no competing interests.

## Authors' contributions

KB, PVA, MM, TP and NDV contributed to the design of the study. KB and LR contributed to the conducting of the study, and monitored recruitment and data collection. KB and PVA performed analyses, and helped with the interpretation of the results and the drafting of the manuscript. All authors helped revise and improve the manuscript, and approved the final manuscript.

## Pre-publication history

The pre-publication history for this paper can be accessed here:

http://www.biomedcentral.com/1471-2458/11/909/prepub
